# Inferior Vena Cava Collapsibility Index: A Precise, Noninvasive Tool for Evaluation of Edema in Children with Nephrotic Syndrome

**DOI:** 10.1007/s12098-022-04177-1

**Published:** 2022-06-17

**Authors:** Hanan El-Halaby, Ashraf Bakr, Mohamed El-Assmy, Hussein Abdelaziz Abdalla, Marwa Salem, Riham Eid

**Affiliations:** 1 Pediatric Intensive Care Unit, Pediatrics Department, Mansoura University Children’s Hospital, Faculty of Medicine, Mansoura University, Mansoura, Egypt; 2 Pediatric Nephrology Unit, Pediatrics Department, Mansoura University Children’s Hospital, Faculty of Medicine, Mansoura University, Mansoura, 35516 Egypt; 3 Biochemistry Department, Faculty of Medicine, Mansoura University, Mansoura, Egypt; 4 Pediatrics Department, Mansoura University Children’s Hospital, Faculty of Medicine, Mansoura University, Mansoura, Egypt

**Keywords:** Children, Nephrotic syndrome, Intravascular volume, Inferior vena cava, Atrial natriuretic peptide, Body composition monitor

## Abstract

This study aimed to evaluate available volume status assessment tools in nephrotic syndrome (NS). Sixty children with INS were subdivided into hypovolemic and nonhypovolemic groups based on fractional excretion of sodium (FeNa%); all were studied for inferior vena cava collapsibility index (IVCCI), plasma atrial natriuretic peptide (ANP), and body composition monitor (BCM). Forty-four patients had nonhypovolemic and 16 had hypovolemic states. ANP did not differ between both groups. IVCCI was higher in hypovolemic group (*p* < 0.001) with sensitivity 87.5% and specificity 81.8% for hypovolemia detection, while BCM overhydration (BCM-OH) values were higher in nonhypovolemic group (*p* = 0.04) with sensitivity = 68.2% and specificity = 75% for detection of hypervolemia. FeNa% showed negative correlation with IVCCI (*r* =  −0.578, *p* < 0.001) and positive correlation with BCM-OH (*r* = 0.33, *p* = 0.018), while FeNa% showed nonsignificant correlation to ANP concentration. IVCCI is a reliable tool for evaluating volume status in NS and is superior to BCM.

## Introduction

There has been a trend to treat edema of idiopathic nephrotic syndrome (INS) children with albumin and furosemide, which is appropriate for hypovolemic patients; however, in non hypovolemic patients, this can precipitate fluid overload. So, it is critical to differentiate types of edema [1]. The currently available tools to assess volume load including inferior vena cava collapsibility index (IVCCI) [2, 3] and bioimpedance spectroscopy (BIS) technique such as body composition monitor (BCM) [4] and plasma atrial natriuretic peptide (ANP) [5, 6]. However, all 3 tools are scarcely evaluated in INS patients and no cutoff levels available. This study was carried out to evaluate the use of IVCCI, BCM, and plasma ANP levels to differentiate type of edema in INS children.

## Material and Methods

Over 3 y, 60 patients were enrolled and classified into hypovolemic and nonhypovolemic groups (nonhypovolemic group was on diuretics at the time of enrollment) based on FeNa%. All investigations were done before any intervention:

For *inferior vena cava collapsibility index (IVCCI) measurement*, the inspiratory and expiratory diameters of the IVC were measured, 2 cm from its entrance into the atrium.

*Body composition monitoring overhydration (BCM-OH)* was done via body composition monitoring (BCM) device (BCM, Fresenius Medical Care D GmbH).

*Plasma atrial natriuretic peptide (ANP) concentration* was measured by enzyme-linked immunosorbent assay (ELISA) technique.

## Results

The detailed clinical, laboratory, and volume status assessment variables for both the groups are presented in Table 1. FeNa% showed negative significant correlation with IVCCI (*r* =  −0.578, *p* < 0.001) and positive significant correlation with BCM-OH (*r* = 0.33, *p* = 0.018), while FeNa% showed nonsignificant correlation to ANP level (*p* = 0.25). To assess the cutoff value of IVCCI, BCM-OH, and plasma ANP concentration that discriminate the volume states in children with active NS, ROC curves for IVCCI, BCM-OH, and serum ANP were plotted against volume state**.** The area under the curve (AUC) for hypovolemia detection for ANP level was nondiscriminative (AUC = 0.511). Correlating clinical histories with IVCCI results showed nonsignificant differences between IVCCI values between patients with SSNS and those with SRNS.

**Table 1:** Clinical and laboratory data of hypovolemic vs. nonhypovolemic patients with active INS at the time of initial enrollment in the study and after resolution of edema

	Hypovolemic patients (*n* = 16)	Nonhypovolemic patients (*n* = 44)	*p*
Age at diagnosis (years)	3.08 ± 1.3	3.8 ± 2.01	0.2
Age at enrollment (years)	6.2 ± 2.5	6.5 ± 2.9	0.76
Duration of illness (month)	39.3 ± 27.4	33.25 ± 28.4	0.46
Sex: male/female	8/8	38/6	**0.003**
Type of NS (clinical situation at enrollment)			** < 0.001**
First attack	2	30	
SDNS	0	4	
FRNS	2	2	
SRNS	12	8	
Degree of edema			0.18
Puffy eyes + LL edema + Ascites	5	20	
As 1 + Scrotal or vulval edema	8	22	
1 or 2 + Pleural effusion	3	2	
Outcome of edema: Resolved	13	43	**0.024**
Nonresolved	3	1	
Percentage of weight reduction until edema resolution (%)	16.9 ± 11.3	11.1 ± 7.04	**0.02**
SBP (mm Hg) at the time of enrollment	105.3 ± 10.5	111.4 ± 12.2	**0.04**
DBP (mm Hg) at the time of enrollment	71.6 ± 14.2	71.05 ± 12.2	0.88
Heart rate (/m) at the time of enrollment	94.2 ± 10.5	89.8 ± 9.7	0.13
Serum albumin (g/dL)	1.95 ± 0.48	1.7 ± 0.26	**0.04**
Serum creatinine (mg/dL)	0.46 ± 0.14	0.58 ± 0.2	**0.024**
Serum Na (mEq/L)	131.4 ± 5.6	137.3 ± 5.4	**0.001**
BUN (mg/dL)	19.4 ± 3.3	16.2 ± 4.1	**0.007**
FeNa (%)	0.16 ± 0.12	1.59 ± 0.67	** < 0.001**
Plasma ANP (pg/mL)	132.97 ± 59.2	127.9 ± 51.7	0.7
Hemoglobin (g/dL)	13.1 ± 1.3	11.1 ± 12	** < 0.001**
Hematocrit (%)	43.8 ± 3.3	37.8 ± 7.9	**0.005**
Expiratory IVC diameter (mm)	9.9 ± 2.9	10.04 ± 1.58	0.8
Inspiratory IVC diameter (mm)	4.7 ± 0.5	6.99 ± 1.52	** < 0.001**
IVCCI on admission	48.97 ± 10.2	30.37 ± 10.98	** < 0.001**
BCM-OH	1.5 (1.95)^*^	2.2 (1.9)^*^	**0.01**

^*^presented as median and interquartile range

Bold values indicate statistically significant values (below 0.05)

*ANP* Atrial natriuretic peptide, *BCM* Body composition monitor, *BUN* Blood urea nitrogen, *DBP *Diastolic blood pressure, *FRNS* Frequent relapsing nephrotic syndrome, *FeNa%* Fractional excretion of sodium, *IVC* Inferior vena cava, *IVCCI* Inferior vena cava collapsibility index, *NS* Nephrotic syndrome, *OH* Over hydration, *SBP* Systolic blood pressure, *SDNS* Steroid-dependent nephrotic syndrome, *SRNS* Steroid-resistant nephrotic syndrome

## Discussion

There are multiple limitations of the use of urinary indices in edema status evaluation in INS, including use of diuretics, antihypertensives, and dietary salt intake [7]. This is why the search is continuous for other simple accurate tools for evaluation of volume status in INS patients.

Brantlov and colleagues reported the valuable use of BCM to distinguish children with active NS from well-treated and healthy children [7], and also Ozdemir et al. [4] reported significant differences in volume burden assessment by BCM between INS children with localized versus those with generalized edema. However, BCM showed moderate sensitivity and specificity for identifying hypervolemia and was nondiscriminative for hypovolemia. BCM can detect the presence and the severity of volume load but cannot define site of extra fluids, and consequently, can be used to monitor response to edema treatment; however, its role in differentiating edema type is limited.

Former studies regarding usefulness of IVCCI in volume evaluation in INS showed contradictory results. In accordance with the results of the present study, Tabel and colleagues observed IVCCI values were significantly increased with diuretic therapy; patients changed from hypervolemia to euvolemic state [8]. Donmez et al. [2] studied the value of IVCCI to determine the volume load in children with minimal lesion NS and noted that IVCCI in edematous patients was less than in nonedematous group and healthy control group, indicating hypervolemic state in edematous patients. To assess the discriminative value of IVCCI as indicator of volume status in active NS, ROC curve was plotted showing a cutoff point 37.5, with an AUC of 0.88, i.e., very good discriminator; more than this point refers to hypovolemic state; and less indicates nonhypovolemia, with sensitivity = 75% and specificity = 86%. However, Ozdemir et al. [4] concluded that using the IVCCI to determine volume load, the sensitivity was 95% and the specificity was 10% and that BIS may be superior to IVCCI in determining volume load in children with INS, which is also against the results of the present study.

Plasma ANP concentration elevation did not reach significant difference between hypovolemic and nonhypovolemic groups. This indicates that ANP plays a role in edema formation in active NS but not in the exact type of edema, and there is some sort of renal resistance to ANP diuretic and natriuretic actions. In NS, resistance to serum ANP is an observation in experimental animals and humans [9, 10], and these studies showed that ANP levels were frequently elevated in INS children as compared to in healthy or remission children.

## Conclusion

Assessment of hydration status in INS children is critical for management of edema. IVCCI is a sensitive and specific tool for discriminating type of edema in INS and is superior to BCM, while serum ANP levels do not seem to be differentiating for the type of edema in INS.

## Data Availability

On request.
